# Intakes of Calcium and Phosphorus and Calculated Calcium-to-Phosphorus Ratios of Older Adults: NHANES 2005–2006 Data

**DOI:** 10.3390/nu7115492

**Published:** 2015-11-19

**Authors:** Reuben Adatorwovor, Kathy Roggenkamp, John J. B. Anderson

**Affiliations:** 1 Departments of Biostatistics, Gillings School of Global Public Health, University of North Carolina, Chapel Hill, NC 27599-7461, USA; radat223@email.unc.edu (R.A.); kathy_roggenkamp@unc.edu (K.R.); 2 Departments of Nutrition, Gillings School of Global Public Health, University of North Carolina, Chapel Hill, NC 27599-7461, USA

**Keywords:** bone mineral density, calcium, phosphorus, calcium:phosphorus ratio, NHANES, RDA

## Abstract

Background: High intakes of dietary phosphorus (P), relative to calcium (Ca) intake, are associated with a lower calcium:phosphorus ratio (Ca:P) ratio which potentially has adverse health effects, including arterial calcification, bone loss, and death. A substantial percentage of older adults (50 to 70 and 71 plus years) who have a higher risk of fracture rate than younger adults typically have low intakes of dietary Ca that are dominated by higher intakes of dietary P from natural and fortified foods, and lower Ca:P ratios than desirable. Objective: This investigation was undertaken to examine Ca and P intakes and the resulting Ca:P ratios (by mass) across gender and older adult age groups, using data from the National Health and Nutrition Examination Survey (NHANES) 2005–2006. Design: NHANES data are based on a cross-sectional sample of the non-institutionalized United States (US) population within various regions. This sample is selected to be representative of the entire US population at all ages. National Cancer Institute (NCI) methods and SAS survey procedures were used for analyses. Ca:P ratios were calculated using total Ca from both foods and supplements, whereas P intakes were calculated from food composition values and supplements. The amounts of P additives in processed foods are not available. Results: Mean Ca and P intakes demonstrated lower intakes of Ca and higher intakes of P compared to current Recommended Dietary Allowances (RDAs). The Ca:P ratios in older male and female adults were influenced by both low-Ca and high-P dietary consumption patterns. Conclusions: Both low total Ca intakes and high P amounts contribute to lower Ca:P ratios, *i.e.*, ~0.7:1.0, in the consumption patterns of older adults than is recommended by the RDAs, *i.e.*, ~1.5:1.0. Whether Ca:P ratios lower than recommended contribute to increased risk of bone loss, arterial calcification, and all-cause mortality cannot be inferred from these data. Additional amounts of chemical P additives in the food supply may actually reduce even further the Ca:P ratios of older adults of both genders, but, without P additive data from the food industry, calculation of more precise ratios from NHANES 2005–2006 data is not possible.

## 1. Introduction

Recent studies of older US adults show that calcium (Ca) intakes from foods and supplements have been close to current recommended dietary allowances (RDAs) for men and women over 50 years of age [1]. Dietary phosphorus (P) intakes, on the other hand, have been shown to greatly exceed current gender-specific RDAs [2]. The intakes of P, however, may be even greater than reported because information on phosphorus food additives is not available to scientists or the public for use in generating total P amounts in the diet [3,4]. As a consequence, only broad estimates of calcium-to-phosphorus (Ca:P) ratios consumed in the US are possible.

Previous investigations generated evidence that a low Ca:P dietary ratio may have an adverse effect on the skeleton because a high P intake leads to a chronically elevated serum parathyroid hormone (PTH) concentration which presumably increases the loss of bone mineral content and density [5,6,7]. Prolonged use of a low Ca:P diet has been considered an important risk factor that contributes to skeletal fractures [6,7,8]. In addition, a high-P diet has been suggested as a risk factor for increased mortality related to cardiovascular disease [9]. High serum P concentration resulting from chronic high P intake has been found to be associated with an increased risk of mortality in individuals with normal renal function [10].

The objective of this analysis is to estimate Ca:P ratios for older American adults, even though the P dietary intake data does not include the amounts of P additives used in food processing. Since high P intakes in typical US diets (3) exceed current RDAs, they drive down the Ca:P ratio and may contribute to adverse health effects, including mortality.

## 2. Methods

In this analysis of dietary intakes of Ca and P according to United States Department of Agricultural (USDA) tables of food composition [11], observations from the 2005–2006 National Health and Nutrition Examination Survey (NHANES) cycle [12] were used. Of the 2214 participants eligible for this study, only 1992 participants remained for the analysis after exclusion of 222 subjects with missing values, zero Ca, zero P, or Ca:P greater than 5.0.

Ca:P ratios were calculated from the NHANES dietary intake and supplement data using mass units for both Ca and P. NHANES used dietary supplement questionnaires for collection of frequency, type, and amount taken in the past 30 days for each supplement for which averages were calculated. Dietary consumption data were collected using two 24-h recalls, one by the mobile examination center and the other by telephone.

We used the National Cancer Institute (NCI) procedures for the primary analysis of the distribution of nutrient intake for Ca, P, and Ca:P ratios. This consists of sets of macros (based on non-linear mixed models) developed to estimate the population distribution of usual intakes from 24-h dietary recalls. Secondary analysis was done using the SAS SURVEYMEANS procedure [13] for the study participants. The DOMAIN statement produced the estimates for age (50–70 and 71+) and gender (male and female) subpopulations [13]. This procedure is primarily a derivative of Taylor expansion, since sampling error estimates are based on complex sample design [14]. Because of the complex sampling design employed by NHANES in obtaining the survey data, weights were used to account for bias (nonresponse) and the unequal probability of sample selection. SAS version 9.4 (TS1M1 SAS Institute Inc., Cary, NC, USA) was used in all analyses. Stratum adjustment for center by NHANES was used in the estimation process.

## 3. Results

**Figure 1:**
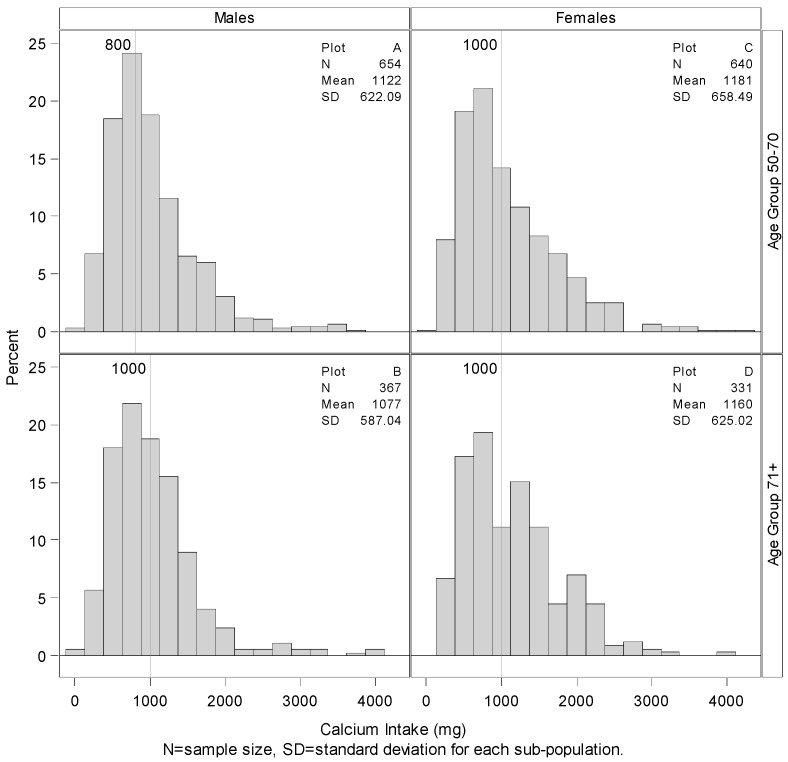
Summary and distributions of total calcium intake with RDAs. Histograms showing the distribution of total calcium intake including supplemental consumption by gender and age group with vertical lines representing RDA amounts. 57.00% of younger males consume more than 800 mg of Calcium per day, 13.00% more than females of the same age group, plots **A** and **C**. Only 42.69% of older males consume more than 1000 mg of calcium per day, 7.61% less than that consumed by older females, plots **B** and **D**.

**Figure 2:**
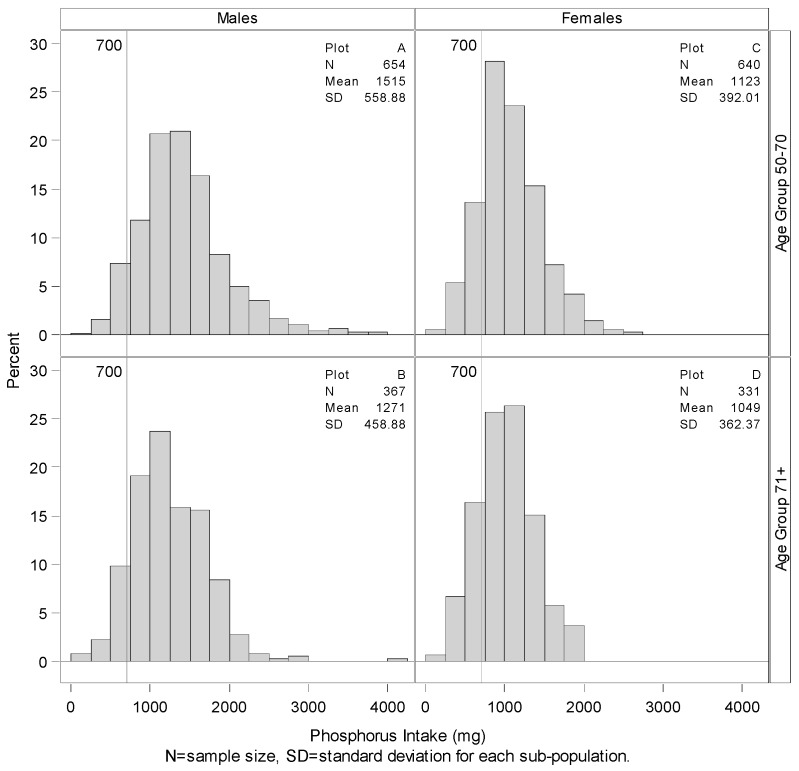
Summary and distributions of total phosphorus intake with RDAs. Histograms showing the distribution of phosphorus by gender and age group with vertical lines representing RDA amounts. Only 1.68%, 3.26%, 6.51% and 8.08% of the younger males, older males, younger females, and older females respectively consume less than the RDA of 700 mg of Phosphorus per day.

**Figure 3:**
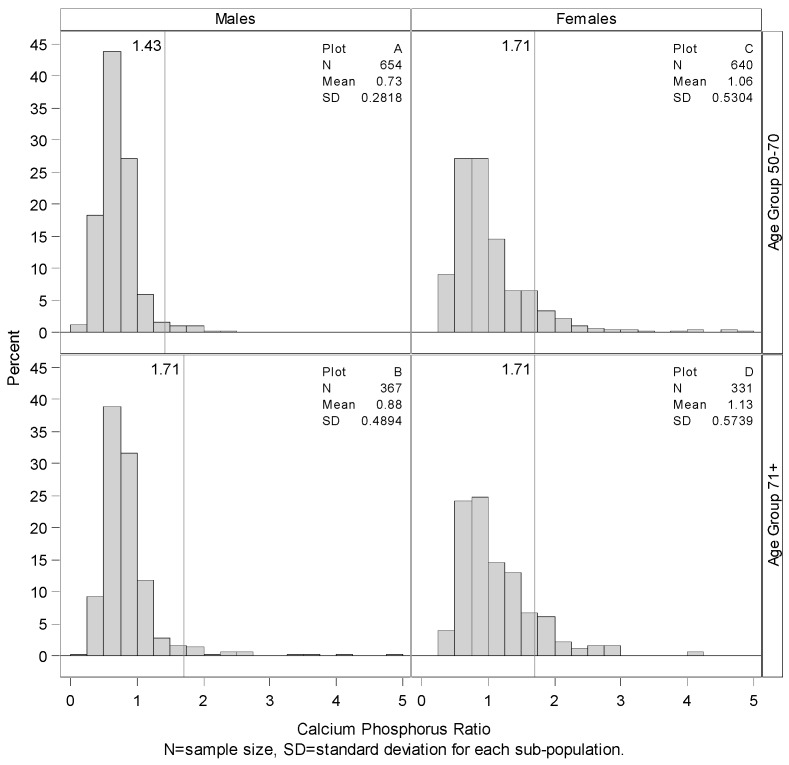
Summary and distributions of estimated Ca:P ratio. Histogram showing the distribution of Ca:P ratio by gender and age group with vertical line representing the mean Ca:P ratio based on Ca and P RDAs (by mass). More than 98.32% of the younger males had Ca:P ratios less than the calculated RDA value of 1.43, plot **A**. Older males had a similar percentage below their calculated RDA-based ratio of 1.71, plot **C**. Among females, 93.64% of the younger group and 92.22% of the older group had a Ca:P ratio lower than the calculated RDA value of 1.71.

Figure 1, Figure 2 and Figure 3 present the estimates of Ca, P, and Ca:P ratio, respectively, across gender and age group. Older adults (71+) for both sexes have lower intakes of Ca and P but a higher Ca:P ratio than the younger groups (Figure 3). Looking at variability, older age groups for both sexes have lower variability for Ca and P but higher variability for Ca:P ratio.

The age- and gender-specific RDAs for each nutrient are shown on Figure 1 and Figure 2. “Recommended” RDAs for Ca:P ratio (by mass) were calculated from the nutrient RDAs for the age and gender groups and are found on Figure 3. Figure 1 shows that only about 57% of younger males consume 800 mg or more of Ca per day, and only 44% of younger females consumed 1000 mg or more of Ca per day. Percentage meeting the daily requirement is even worse for the older adults. Only about 43% of older males consume 1000 mg or more of Ca per day compared to about 51% of older females. Figure 2 shows that only 1.68%, 3.26%, 6.51% and 8.08% of the younger males, older males, younger females, and older females respectively consume 700 mg or less of P per day compared to the RDA estimate.

Sensitivity analyses were conducted, first with removal of all the extreme values (values such as zero grams of Ca and values of Ca or P greater than 4500 mg per day), and second, with the data in moles instead of grams. In both analyses, our conclusions and findings were consistent with the primary analysis as Ca:P ratio remained relatively unchanged. These results were much worse when we considered only dietary Ca intake without any supplements.

Additionally, we used the SAS SURVEYMEANS procedure to analyze the within person variability among variables and found that the results were highly consistent with our findings. The mean estimates were exactly the same with just the variability for each age group and gender slightly lower for Ca consumption and P consumption and mostly identical for Ca:P ratio.

## 4. Discussion

Females within both age groups (50–70 and 71+) had higher mean total Ca intakes, including supplements, than males (Figure 1). These findings contrasted with lower P intakes by females than by males in both age groups. Based on gender intake differences, higher Ca:P ratios were found for women than men in both older adult groups.

Whether the observed higher Ca:P ratios of older females, compared to younger females, contribute to healthier bone mineral density (BMD) measurement and potentially lower fracture rates have not been demonstrated. The lower dietary Ca:P ratios in older men compared to older women seem to have little or no bearing on risk of fractures or death, but the long-term prospective effects of low Ca:P ratios in men and women have not been investigated.

Supplemental Ca consumption which was much greater in older women of both age groups than for men is the major reason for the higher mean Ca:P ratios, but to a lesser degree the consumption of foods with greater Ca content also contributed. Older men consumed more P-rich foods, especially meats, than older women. However, fracture rates resulting from low bone mass are known to be much lower in males than females of both age groups, despite greater consumption of additive phosphate salts used in meats and other foods by men. Based on this finding, Ca:P ratio seems to have limited utility for assessing risk of skeletal fractures in old age.

Concerns about excessive P intakes extend beyond the potential adverse skeletal effects of a persistently elevated parathyroid hormone concentration [8]. The complex homeostatic mechanisms regulating serum Ca and P concentrations may be significantly challenged by chronically high intakes of P. Repetitive hormonal adaptations relating to elevated post-meal serum concentrations of phosphate ions may signal cells to calcify in pathologically damaged arterial and heart valvular walls.

Vascular calcification may result from excessive dietary P consumption from foods and food additives, as demonstrated in cell models [15,16,17,18,19,20,21]. Our results provide no insight on this phenomenon, but high P intake patterns, especially those ingesting more than 1400 mg per day, have been shown to increase all-cause mortality of otherwise healthy US adults in an analysis of NHANES III data, perhaps in part because of arterial calcification [9] and in general [10]. Several chronic diseases are considered to be affected by high P intakes, although mechanisms have not been established [22].

A major limitation of this study is that no causal effect of a low Ca:P ratio on adverse health effects can be established. Rather, a potential sequence of associations can be drawn from the data analyses presented: excessive phosphorus intakes, inadequate calcium in the diet, or low Ca:P ratio may contribute to higher levels of serum phosphate concentration, post-intake increases of PTH secretion, increased risks of vascular pathology, a decline in bone mass and strength, and increased mortality of both men and women, even among those who are generally considered healthy. Stratified analyses for race and economic status were not performed on this representative sample of elders. We assumed that all participants were exposed to natural foods that contains phytate, a partial inhibitor of intestinal Ca absorption. In addition, the results of this analysis would change if RDA values for either or both Ca and P were revised by the Institute of Medicine (IOM).

Prospective randomized controlled trials of experimental subjects fed diets containing different Ca:P ratios are needed to clarify the concerns about potential adverse skeletal effects and fractures resulting from the consumption of low Ca:P ratio diets. Additional research is needed to examine the relationship between Ca:P ratio and potential health effects on bone mineral density.

## 5. Conclusions

Ca:P ratios based on food consumption patterns of older males and females illustrate the wide variability of these two micronutrients used for maintaining bone health and other functions. The intake ratios are not in congruence with Ca:P ratios, *i.e.*, ~2:1, based on recommended intakes of Ca and P, according to the current RDAs. Because of widespread use of calcium supplements, older women, in both the 50–70 and 71-plus year age range, have higher Ca:P ratios than men of both age groups. Adults have missed attaining the recommended intakes of Ca and P across older age groups and gender. As a result, they tend to have low Ca:P ratios. Whether these lower Ca:P ratios have adverse effects regarding bone fracture or vascular calcification remains unclear and requires further investigation. Lowering P intake is clearly recommended for older adults so that any of these potential risks of adverse effects of low Ca:P ratios, including mortality, may be reduced.
